# Genetic regulatory mechanisms in human osteoclasts suggest a role for the *STMP1* and *DCSTAMP* genes in Paget’s disease of bone

**DOI:** 10.1038/s41598-018-37609-0

**Published:** 2019-01-31

**Authors:** Benjamin H. Mullin, Kun Zhu, Suzanne J. Brown, Shelby Mullin, Jennifer Tickner, Nathan J. Pavlos, Frank Dudbridge, Jiake Xu, John P. Walsh, Scott G. Wilson

**Affiliations:** 1 Department of Endocrinology & Diabetes, Sir Charles Gairdner Hospital, Nedlands, WA Australia; 2 School of Biomedical Sciences, University of Western Australia, Crawley, WA Australia; 3 Medical School, University of Western Australia, Crawley, WA Australia; 4 Department of Health Sciences, University of Leicester, Leicester, UK; 5 Department of Twin Research & Genetic Epidemiology, King’s College London, London, UK

## Abstract

Paget’s disease of bone (PDB) is characterised by focal abnormalities of bone remodelling, with increased osteoclastic resorption the primary feature of the disease. Genetic factors have been shown to play an important role in PDB, and genome-wide association studies (GWAS) have identified 7 genetic loci as associated with PDB at the genome-wide level. Expression quantitative trait locus (eQTL) studies using cell types that are directly relevant to the disease of interest are increasingly being used to identify putative effector genes for GWAS loci. We have recently constructed a unique osteoclast-specific eQTL resource using cells differentiated *in vitro* from 158 subjects for study of the genetics of bone disease. Considering the major role osteoclasts have in PDB, we used this resource to investigate potential genetic regulatory effects for the 7 PDB genome-wide significant loci on genes located within 500 kb of each locus. After correction for multiple testing, we observed statistically significant associations for rs4294134 with expression of the gene *STMP1*, and rs2458413 with expression of the genes *DPYS* and *DCSTAMP*. The eQTL associations observed for rs4294134 with *STMP1*, and rs2458413 with *DCSTAMP* were further supported by eQTL data from other tissue types. The product of the *STMP1* gene has not been extensively studied, however the *DCSTAMP* gene has an established role in osteoclast differentiation and the associations seen between rs2458413 and PDB are likely mediated through regulatory effects on this gene. This study highlights the value of eQTL data in determining which genes are relevant to GWAS loci.

## Introduction

Paget’s disease of bone (PDB), first described by Sir James Paget in 1876, is characterised by focal abnormalities of bone remodelling. It is the most common metabolic bone disease after osteoporosis, affecting around 2% of European individuals aged 55 years and over^1^, although both the incidence and severity of newly diagnosed cases is falling^2,3^. The prevalence of PDB increases with age and is slightly more common in men than in women. The abnormal bone remodelling seen in PDB disrupts normal bone architecture and structure, leading to deformity, bone pain, secondary arthritis and increased fracture risk^4^. The primary feature of PDB at the cellular level is increased osteoclastic bone resorption, followed by increased and disorganised bone formation that is thought to be secondary to the increased osteoclast activity. The osteoclasts in affected bone are larger in size, have more nuclei and display characteristic intra-nuclear inclusion bodies.

Genetic factors have been shown to play an important role in the pathogenesis of PDB and are generally considered to be the primary risk-factor for developing the disease, with relatives of an affected individual having around 7-fold greater risk of developing PDB than the general population^5^. The genetic architecture of the disease is complex, with contributions from rare mutations in the *SQSTM1* gene as well as common variants with small effects located in other genes. Mutations in *SQSTM1* have been found to cause autosomal dominant PDB with high but incomplete penetrance^6–8^, and may account for up to 40% of familial PDB cases and 10% of apparently sporadic PDB cases^7,9^. The common non-*SQSTM1* PDB risk-variants are thought to contribute to the disease in an additive manner and interact with *SQSTM1* mutations to influence disease severity^10^.

Genome-wide association studies (GWAS) have successfully identified many of the common genetic variants associated with PDB^11,12^. The most recent of these, performed by Albagha *et al*.^12^ in a study cohort of 2,215 PDB cases without *SQSTM1* mutations and 4,370 controls, identified 7 loci as associated with PDB at the genome-wide significance level. Six of the 7 maximally associated variants at these loci are non-coding in nature, located in intronic or intergenic DNA, suggesting that they may have regulatory effects on the expression of nearby genes. Although some strong candidates have been identified, the identity of many of the genes regulated by these GWAS loci is yet to be determined.

Expression quantitative trait locus (eQTL) studies have increasingly been used to identify putative effector genes for GWAS loci by characterising association between genetic variants and the expression of nearby genes. However, complex tissues containing a mixture of multiple cell types have been used for many of these studies and, apart from a study in osteoblasts derived from trabecular bone explants^13^, there has historically been a lack of information specifically relevant to bone cells. We have recently constructed a unique osteoclast-specific eQTL dataset for study of the genetics of bone disease^14^. Considering the major role osteoclasts have in PDB, we sought to use this resource to investigate potential genetic regulatory effects for the 7 genome-wide significant loci for PDB.

## Results

The demographics of the study cohort are presented in Supplementary Table S1. None of the 158 study participants were found to be closely related (all PI_HAT < 0.1) and principal components analysis of the unimputed genotype data did not detect any outliers. After imputation and QC procedures had been applied, there were 5,373,348 variants in the genotype dataset with an IMPUTE2 info score ≥0.4 and a minor allele frequency (MAF) ≥5%. In the RNA-Seq dataset derived from the differentiated osteoclast-like cells, quantitative expression data was obtained for 15,688 genes. Particularly high expression was observed for several osteoclast marker genes, including *ACP*5, *ATP6V0D2*, *CA2*, *CTSK*, *DCSTAMP*, *MMP9*, *OCSTAMP* and *SPP1* (Supplementary Figure S1), thereby confirming the appropriateness and rigor of the culture protocol.

### Association of PDB GWAS variants with gene expression

**Figure 1:**
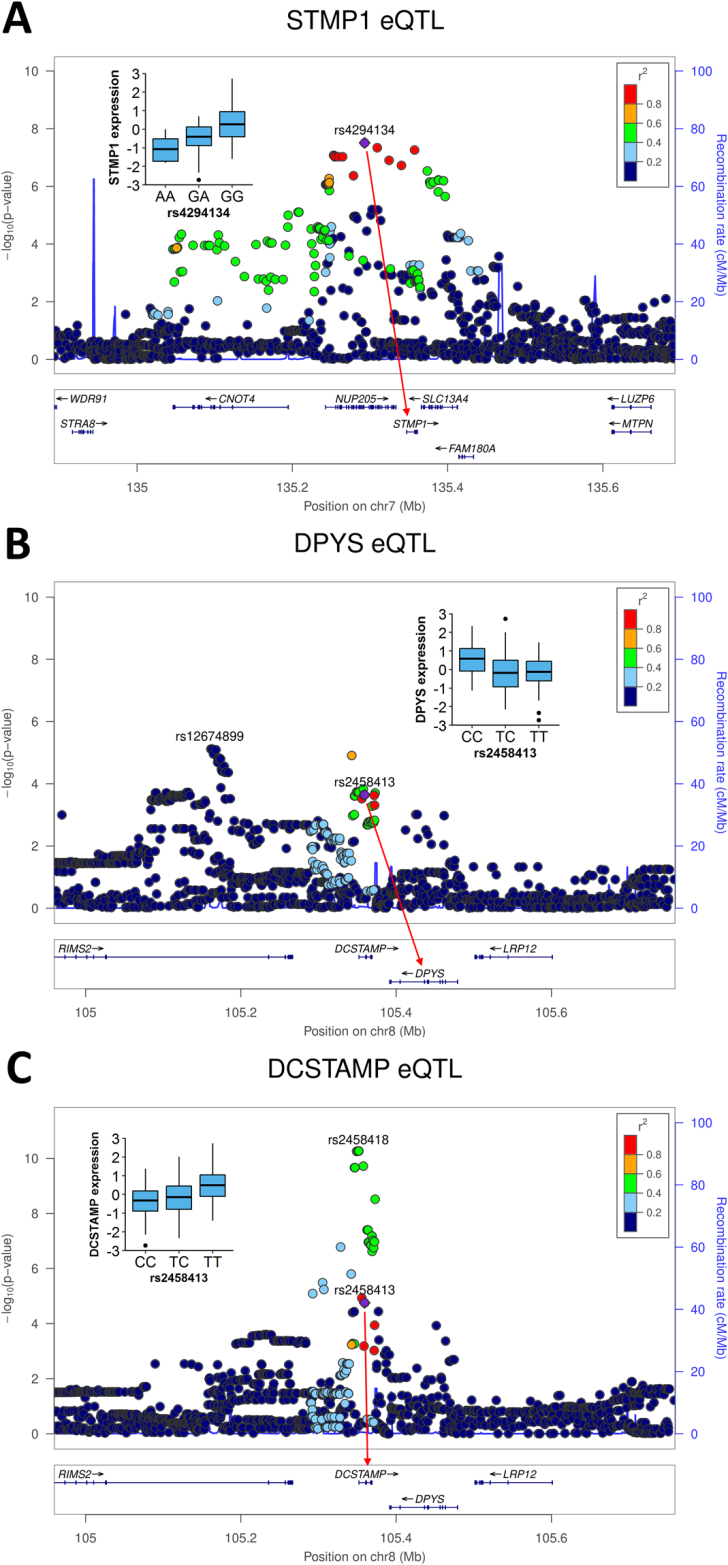
Regional association plots generated using the eQTL association results for (**A**) *STMP1*, (**B**) *DPYS* and (**C**) *DCSTAMP* from the osteoclast-specific dataset. Genetic variants within 400 kb of rs4294134 or rs2458413 are depicted (x axis) along with their eQTL *P* value (−log10). For *DPYS* and *DCSTAMP*, the maximally associated eQTL variant from the region is also indicated (rs12674899 and rs2458418, respectively). Variants are colour coded according to their LD (r^2^) with PDB GWAS loci rs4294134 or rs2458413 (1000GP Nov 2014 EUR population). The red arrows indicate significant associations between rs4294134 or rs2458413 and expression of nearby genes, with the allelic effects on normalised gene expression presented in the box-and-whisker plots. The recombination rate (blue line) and position of genes, their exons and direction of transcription is also indicated^32^.

We analysed the 7 variants identified as significantly associated with PDB at the genome-wide level by Albagha *et al*.^12^, rs10494112 (1p13.3), rs4294134 (7q33), rs2458413 (8q22.3), rs1561570 (10p13), rs10498635 (14q32.12), rs5742915 (15q24.1) and rs3018362 (18q21.33), for association with local gene expression (+/−500 kb) in our osteoclast eQTL dataset. All 7 variants were found to have a MAF ≥5% in the eQTL dataset, with a total of 56 expressed gene transcripts identified within the combined analysis windows (mean 8 per variant). After correction for multiple testing within each locus, we observed statistically significant associations for rs4294134 with expression of the gene *STMP1* (Table 1 and Fig. 1A), and rs2458413 with expression of the genes *DPYS* and *DCSTAMP* (Table 1 and Fig. 1B,C). For rs4294134, the PDB risk-increasing *G* allele was associated with increased expression of *STMP1* (Table 1). For rs2458413, the PDB risk-increasing *T* allele was associated with reduced expression of *DPYS* and increased expression of *DCSTAMP* (Table 1).

**Table 1:** PDB GWAS variants demonstrating significant eQTL associations in the osteoclast-like cells.

Variant	Location	EA	OA	EAF	PDB OR (95% CI)	Gene	Expression^a^	Distance to TSS	P	Beta^b^
rs4294134	chr7:135608380	G	A	0.82	1.45 (1.29–1.63)	*STMP1*	10.44 ± 1.71	−54,116	3.11E-08	0.739
rs2458413	chr8:104347204	T	C	0.56	1.40 (1.29–1.51)	*DPYS*	0.21 ± 0.16	−119,850	2.27E-04	−0.396
rs2458413	chr8:104347204	T	C	0.56	1.40 (1.29–1.51)	*DCSTAMP*	110.75 ± 43.60	8,117	1.76E-05	0.468

EA: effect allele; OA: other allele; EAF: effect allele frequency (derived from osteoclast eQTL cohort); PDB: Paget’s disease of bone; OR: odds ratio; CI: confidence interval; TSS: transcription start site; variant locations obtained from dbSNP build 150 (GRCh38/hg38); PDB odds ratios obtained from Albagha *et al*.^12^. The eQTL associations are statistically significant using a multiple testing corrected FDR of 5% (analysis corrected for the covariates RNA-Seq batch, patient age and 10 principal components).

^a^Expression levels are stated as mean reads per kilobase million (RPKM) ± standard deviation.

^b^Normalised effect size on gene expression for the effect allele.

### Top eQTL variants for the *STMP1*, *DPYS* and *DCSTAMP* genes

We next examined all variants located within +/−500 kb of the *STMP1*, *DPYS* and *DCSTAMP* transcription start sites (TSS) to identify the top *cis*-eQTL variant for each gene. For *STMP1*, the PDB GWAS variant rs4294134 was found to demonstrate the strongest association with expression of the gene (Fig. 1A). For the *DPYS* gene, rs12674899 was identified as the top eQTL variant from the region, with the more common *G* allele associated with increased expression of the gene (*P* = *7.67* × *10*^−*6*^, Fig. 1B). This variant was found to exhibit a low level of linkage disequilibrium (LD) with the PDB GWAS variant rs2458413 (r^2^ = 0.13), suggesting that the two may represent independent eQTL association signals. For the *DCSTAMP* gene, rs2458418 was identified as the maximally associated eQTL variant in the region, with the more common *C* allele associated with increased expression of the gene (*P* = *5.36* × *10*^−*11*^, Fig. 1C). This variant was found to exhibit a moderate degree of LD with the PDB GWAS variant rs2458413 (r^2^ = 0.43), suggesting that the two may be part of the same eQTL association signal.

### Replication in other cell/tissue types

The 2 PDB GWAS/eQTL variants identified in this study, rs4294134 and rs2458413, were queried using the publicly available GTEx Portal^15^ for evidence of eQTL effects in other tissues. These variants were checked against pre-calculated eQTLs from the GTEx Portal, which were identified for tissues with ≥70 samples using a q-value threshold of 0.05 corrected for multiple-testing. Supporting evidence for regulatory effects was found for rs4294134 with the gene *STMP1* in 38 tissue types (*P* = *4.5* × *10*^−*6*^ – *6.6* × *10*^−*42*^) and rs2458413 with *DCSTAMP* in lung tissue (*P* = *1.1* × *10*^−*10*^). For all of these significant associations, the allelic effect seen for rs4294134 and rs2458413 on gene expression was consistent with that observed in this study of osteoclasts.

The top eQTL variants identified for the *DPYS* and *DCSTAMP* genes in this study, rs12674899 and rs2458418, were also queried using the GTEx Portal for evidence of replication. No significant associations were seen between the variant rs12674899 and expression of *DPYS* in any of the GTEx tissues. However, the variant rs2458418 was found to be significantly associated with expression of the *DCSTAMP* gene in 5 tissue types (*P* = *2.0* × *10*^−*5*^ – *6.8* × *10*^−*45*^), and demonstrated the second strongest association of all variants in the region behind rs2514662, with which it is in strong LD (r^2^ = 0.97). For each of these tissues, the more common *C* allele for rs2458418 was associated with higher *DCSTAMP* expression, consistent with the allelic effect observed in this study.

### Bioinformatics analysis

Analysis of rs4294134 using HaploReg v4.1^16^ revealed that it is in strong LD (r^2^ ≥ 0.8) with 10 other variants in the region. Of these, the variant rs6467603 is a strong candidate for having a regulatory role it lies within a variety of predicted regulatory elements, including promoter histone marks (8 tissues), enhancer histone marks (15 tissues), DNase hypersensitivity sites (10 tissues) and 7 regulatory motifs. The variant rs3110823 also presents strongly from a regulatory perspective, being located in promoter histone marks (2 tissues), enhancer histone marks (13 tissues), DNase hypersensitivity sites (9 tissues), 3 regulatory motifs and a GERP (Genomic Evolutionary Rate Profiling) and SiPhy conserved region.

Analysis of rs2458413 suggested that it is in strong LD (r^2^ ≥ 0.8) with 6 other variants, of which rs2669448 may have a potential regulatory role. The variant is located within several predicted regulatory elements, including a promoter histone mark (1 tissue), enhancer histone marks (10 tissues), DNase hypersensitivity sites (10 tissues), and is predicted to alter 2 regulatory motifs.

## Discussion

We have used our unique osteoclast-specific eQTL dataset to identify a potential regulatory role for the PDB GWAS variant rs4294134 on expression of the gene *STMP1* (*short transmembrane mitochondrial protein 1*). This finding was strongly supported by publicly available eQTL data in multiple tissue types from the GTEx Portal^15^. The *G* allele at rs4294134, which is associated with an increased risk of PDB^12^, was found to be associated with increased expression of *STMP1* in our osteoclast eQTL dataset, consistent with the allelic effect observed in the GTEx tissues. The fact that rs4294134 demonstrated the strongest association with expression of *STMP1* of all tested variants in our dataset situated within 500 kb of the gene TSS strongly suggests that the association between this variant and PDB is mediated through regulatory effects on *STMP1*. The product of the *STMP1* gene is a short trans-membrane protein that may have a role as a mitochondrial respiratory complex subunit^17^. This gene has not been extensively studied and has no obvious role in bone biology, although knockdown of the *STMP1* gene in zebrafish resulted in a series of mild morphological defects, including abnormal shape of the head and jaw^17^. Albagha *et al*.^12^ initially suggested that the association between rs4294134 and PDB may be mediated through the gene *NUP20*5, which encodes one of the main subunits of the nuclear pore complex involved in the regulation of transport between the cytoplasm and nucleus^18^. However, that was based primarily on the location of rs4294134 within *NUP205* and not on functional evidence, with the authors acknowledging that no genes in the region were known to affect bone metabolism. Our data showed no evidence of a role for rs4294134 in regulating expression of *NUP205* in this cell type.

We have identified an eQTL association between the PDB GWAS variant rs2458413 and expression of the gene *DPYS* in this study. A potentially independent eQTL association signal relevant to the *DPYS* gene and led by rs12674899 also was identified in the region. The *DPYS* gene encodes the enzyme dihydropyrimidinase, which has a role in pyrimidine metabolism. Mutations in the *DPYS* gene have been implicated in dihydropyrimidinase deficiency (OMIM #222748), a very rare autosomal recessive condition characterised by accumulation of dihydrouracil and dihydrothymine in the urine, blood and cerebrospinal fluid. Clinical features of this condition are highly variable and primarily include gastrointestinal abnormalities, hypotonia, seizures and neurological abnormalities^19^. The product of the *DPYS* gene has no obvious role in bone biology and it should be noted that this gene was expressed at a relatively low level in the osteoclast-like cells, suggesting that the eQTL association between rs2458413 and expression of *DPYS* may be more relevant in other cell types and disorders unrelated to PDB.

The PDB GWAS variant rs2458413 was also identified as significantly associated with expression of the *DCSTAMP* (*dendrocyte expressed seven transmembrane protein*) gene in this study. The PDB risk-increasing *T* allele at rs2458413 was found to be associated with increased expression of *DCSTAMP*, consistent with the allelic effect observed for this gene in lung tissue from the GTEx Portal. The product of the *DCSTAMP* gene is a multi-pass transmembrane protein that is considered to be a master regulator of osteoclastogenesis, as it is essential for the cell fusion that occurs between osteoclast precursors. Kukita *et al*.^20^ demonstrated that expression of the murine *Dcstamp* gene is upregulated in mouse osteoclast precursors when cultured in the presence of the osteoclastogenic cytokine receptor activator of nuclear factor kappa-B ligand (RANKL). They also found that knockdown of *Dcstamp* inhibited the formation of multinucleated mouse osteoclast-like cells, while overexpression enhanced osteoclastogenesis in the presence of RANKL. Yagi *et al*.^21^ subsequently demonstrated that *Dcstamp*-knockout mice display an osteopetrotic phenotype due to an absence of functional multinucleated osteoclasts. In humans, osteoclast-like cells derived from PDB patients that are carriers for a rare non-synonymous coding variant in the *DCSTAMP* gene (rs62620995) present with increased nuclei number compared to cells derived from healthy controls^22^. These cells also demonstrated increased expression of the *DCSTAMP* gene compared to those obtained from PDB patients that do not carry the variant^22^. In the study by Albagha *et al*.^12^, *DCSTAMP* was identified as the likely effector gene from the PDB genome-wide significant locus at 8q22.3. Our data supports this by suggesting that the PDB GWAS variant rs2458413 is exerting an effect on PDB susceptibility though regulatory effects on the *DCSTAMP* gene. Considering the established role of *DCSTAMP* in osteoclastogenesis, it seems likely that increased expression of *DCSTAMP* leads to enhanced osteoclast fusion, thereby contributing to the focal lesions of PDB.

There are some potential limitations to this study. One of these is that the osteoclast-like cells used in these experiments were cultured and differentiated *in vitro*, and therefore potentially exhibit somewhat different gene expression profiles to osteoclasts *in vivo* which are subject to the local bone microenvironment. Other potential limitations include the difficulties associated with using cells derived from the circulatory system to study a disease like PDB with a focal nature, as well as the fact that the eQTL effects identified in this study could be more relevant to females due to the absence of males in the study cohort. It should also be noted that the research materials in this study were not focused on individuals with PDB. However, considering the complex nature of the genetics of PDB and the fact that these PDB GWAS variants have a common MAF in the general population, we consider that our findings are relevant and representative of true contributing factors to both osteoclast biology in general and also to this disease. In the case of *DCSTAMP*, it is possible that increased expression of this gene has minimal effects in the general population, but in individuals at increased risk of PDB due to environmental or other genetic factors the increased expression facilitates enhanced fusion of osteoclast precursors and subsequently formation of the abnormally large, overactive osteoclasts that are characteristic of the disease.

In conclusion, we have identified regulatory effects for the PDB GWAS variants rs4294134 (*STMP1*) and rs2458413 (*DPYS* and *DCSTAMP*) in human osteoclast-like cells. Our data do not support a role for rs4294134 in the regulation of *NUP205* in this cell type, as has been proposed previously. The eQTL associations observed for rs4294134 with *STMP1*, and rs2458413 with *DCSTAMP* were supported by publicly available eQTL data from other tissue types. The *STMP1* gene has not been extensively studied to date, however the *DCSTAMP* gene has an established role in osteoclast differentiation and the associations seen between rs2458413 and PDB are likely mediated through regulatory effects on this gene.

## Methods

### Subject recruitment

Recruitment of participants and laboratory procedures has been previously described^14^. In brief, 158 female participants aged 30–70 were recruited for the study. These individuals had been referred for dual-energy X-ray absorptiometry (DXA) BMD scanning (Hologic, Bedford, MA, USA) at the Bone Density Unit at Sir Charles Gairdner Hospital in Western Australia. Only females were recruited for the study to control for gender-specific differences in osteoclast gene expression. Patients completed a medical history questionnaire, with a number of exclusion criteria applied including the presence of medical conditions or use of medications likely the influence the ability of their cells to differentiate into osteoclasts or resorb bone. To minimise potential issues with population stratification, only subjects with self-reported European ancestry were included in the study. A blood sample was obtained from each patient, which consisted of two 6 ml lithium heparin and one 4 ml ethylenediaminetetraacetic acid (EDTA) sample. Approval for the study was granted by the Sir Charles Gairdner and Osborne Park Health Care Group Human Research Ethics Committee and all participants provided written informed consent. The study was performed in accordance with applicable regulations and guidelines.

### Isolation of peripheral blood mononuclear cells and osteoclastogenesis

The lithium heparin blood tubes obtained from each patient were used for isolation of peripheral blood mononuclear cells (PBMCs) by density gradient centrifugation as described previously^14^ using well-established protocols in our laboratory^23^. Triplicate cultures were grown for each sample in 24-well cell culture plates, with each well seeded with 1.5 × 10^6^ cells suspended in complete α-MEM (minimum essential medium) supplemented with 25 ng/ml macrophage colony stimulating factor (M-CSF). After 2 days, the culture medium was replaced with complete α-MEM supplemented with 25 ng/ml M-CSF and 100 ng/ml RANKL. The cells were grown in this medium formulation for another 12 days, during which time they underwent osteoclastogenesis. We have previously demonstrated that osteoclast-like cells cultured using this protocol are capable of resorbing bone in an *in vitro* pit assay^14^. Staining of the differentiated cells for tartrate resistant acid phosphatase (TRAP) was performed as an additional characterisation of the osteoclast phenotype.

### Nucleic acid extraction

The QIAamp DNA Blood Mini Kit (QIAGEN) was used to extract genomic DNA from 200 μl of each EDTA blood sample. Each set of triplicate osteoclast-like cell cultures was harvested at day 14 of culture and combined into a single aliquot before genomic DNA and RNA were extracted using the AllPrep DNA/RNA Mini Kit (QIAGEN). An on-column DNase digestion step was performed for the RNA fraction of each sample to prevent DNA contamination of the RNA samples. Each RNA sample was subjected to quality assessment using the Agilent 2100 Bioanalyzer, with all samples generating RNA integrity numbers (RINs) ≥9.7.

### Genotyping and imputation

Each genomic DNA sample extracted from EDTA blood was subjected to genotyping using the Illumina Infinium OmniExpress-24 BeadChip array. Pre-imputation QC criteria were applied to the genotype data, including removal of individuals and variants with a call rate <90%, removal of variants that were monomorphic, unmapped, had a MAF <5% or Hardy-Weinberg equilibrium *P* < *5* × *10*^−*8*^. After these QC criteria had been applied, 572,898 variants remained in the genotype dataset. The Sanger Imputation Service was then used to perform genotype imputation using the Haplotype Reference Consortium (HRC) release 1.1 reference panel^24^. Post-imputation QC included removal of variants with an IMPUTE2 info score <0.4.

### Relatedness testing and principal components analysis

Principal components analysis and identity-by-descent (relatedness) testing were performed on the genotype data using Plink v1.9^25^. We generated 10 principal components for the study cohort, which were used to check for outliers and were included as covariates in the eQTL analysis to correct for any population stratification that may be present.

### Gene expression analysis

Gene expression was quantitated in the RNA samples extracted from the osteoclast-like cells using 50 bp single-end RNA-Seq as described previously^14^. Trimmed mean of M-values (TMM) normalisation and correction of the data for total read count by conversion to counts per million (CPM) was performed using the edgeR package^26^. This software was also used to calculate reads per kilobase million (RPKM) values to allow comparison of expression levels between different genes and with other tissues.

### eQTL analysis

The TMM normalised CPM values were used for the eQTL analysis, which was performed using the FastQTL software^27^. This software efficiently implements linear regression to identify associations between genotypes and gene expression phenotypes. A hypothesis-driven association analysis was performed for the quantile-normalised gene expression levels (generated using the *rntransform* function of the GenABEL package^28^) with imputed genotype probabilities for the 7 PDB GWAS variants. The covariates RNA-Seq batch, patient age and 10 principal components were included in the eQTL analysis. All expressed genes with a TSS located within 500 kb of a PDB GWAS variant were included in the analysis (*cis-*eQTLs). Each locus was corrected for multiple testing using the Benjamini-Yekutieli procedure^29^ with a false discovery rate (FDR) of 5%.

### Bioinformatics analysis

HaploReg v4.1^16^ and RegulomeDB^30^ were used to perform bioinformatics analysis of individual eQTL-variants to identify other variants in strong LD and to characterise potential regulatory effects. Analysis of LD between eQTL variants within the same locus was performed using LDlink (1000 G Phase 3 EUR population)^31^.

## Supplementary information

Supplementary Information

## Data Availability

The osteoclast eQTL data described in this study relevant to the *STMP1*, *DPYS* and *DCSTAMP* genes are available online at https://research-repository.uwa.edu.au/.
