# Supplementary material for: Genetic regulatory mechanisms in human osteoclasts suggest a role for the STMP1 and DCSTAMP genes in Paget’s disease of bone
Source: Sci Rep. 2019 Jan 31;9:1052. doi: 10.1038/s41598-018-37609-0 (PMC6355970; doi:10.1038/s41598-018-37609-0)
Supplement: Supplementary file 1 — Supplementary Information [file 41598_2018_37609_MOESM1_ESM.pdf]

Genetic regulatory mechanisms in human osteoclasts suggest a role for the *STMP1* and *DCSTAMP* genes in Paget's disease of bone

Benjamin H Mullin[1,2*], Kun Zhu[1,3], Suzanne J Brown[1], Shelby Mullin[1,2], Jennifer Tickner[2], Nathan J Pavlos[2], Frank Dudbridge[4], Jiake Xu[2], John P Walsh[1,3], Scott G Wilson[1,2,5]

[1]Department of Endocrinology & Diabetes, Sir Charles Gairdner Hospital, Nedlands, WA, Australia

[2]School of Biomedical Sciences, University of Western Australia, Crawley, WA, Australia

[3]Medical School, University of Western Australia, Crawley, WA, Australia

[4]Department of Health Sciences, University of Leicester, Leicester, UK

[5]Department of Twin Research & Genetic Epidemiology, King's College London, London, UK

Supplementary Information

Supplementary Table S1. Demographics of the study cohort

Demographic variable	Mean ± SD
Subjects (n)	158
Age (years)	57.2 ± 9.7
Age (range)	30.5 – 69.8
Height (cm)	162.7 ± 6.2
Weight (kg)	67.7 ± 13.0
BMI (kg/m^2)	25.6 ± 5.1
Spine BMD (g/cm^2)	0.94 ± 0.15
Spine BMD T-score	-0.96 ± 1.34
Total hip BMD (g/cm^2)	0.84 ± 0.12
Total hip BMD T-score	-0.80 ± 0.99
Femoral neck BMD (g/cm^2)	0.72 ± 0.11
Femoral neck BMD T-score	-1.17 ± 0.96

SD: standard deviation; BMD: bone mineral density.

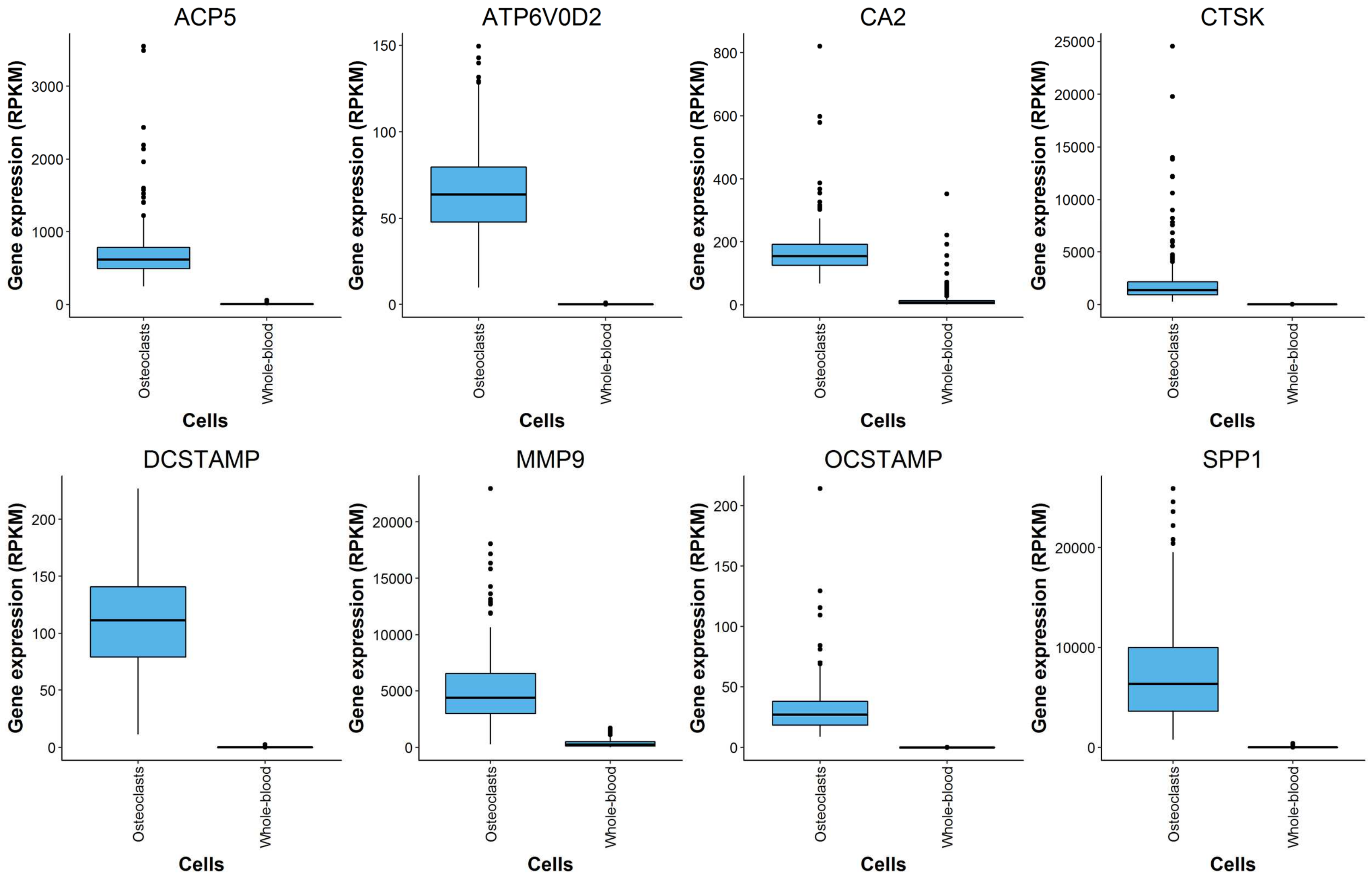

Supplementary Fig. S1. Box-and-whisker plots displaying expression levels of several osteoclast marker genes in the osteoclast-like cells relative to the GTEx whole-blood sample set (n=393).
